# Revitalizing Hands: A Comprehensive Review of Anatomy and Treatment Options for Hand Rejuvenation

**DOI:** 10.7759/cureus.35573

**Published:** 2023-02-28

**Authors:** Lior Har-Shai, Sar-El Ofek, Tomer Lagziel, Yoav Y Pikkel, Ori S Duek, Dean D Ad-El, Tamir Shay

**Affiliations:** 1 Department of Plastic Surgery & Burns, Rabin Medical Center – Beilinson Hospital, Petach Tikva, ISR; 2 Sackler Faculty of Medicine, Tel-Aviv University, Tel-Aviv, ISR; 3 Department of Plastic and Reconstructive Surgery, Rambam Medical Center, Haifa, ISR; 4 The Ruth and Bruce Rappaport Faculty of Medicine, Technion-Israel Institute of Technology, Haifa, ISR

**Keywords:** dermal fillers, fat grafting, hand aging, hand anatomy, skin aging, injectables, aesthetic medicine, hand rejuvenation

## Abstract

Dorsal hand rejuvenation is gaining popularity as a solitary procedure and adjunct to face and neck rejuvenation treatments. As the hands age, the skin loses elasticity and becomes more translucent, the veins, joints, and tendons appear more prominent, and the bones become more noticeable. These changes are due to intrinsic and extrinsic factors. Current treatment methods include the injection of dermal fillers and autologous fat grafting. Anatomic studies to ensure the successful implementation of rejuvenation procedures identified three separate fascial layers in the dorsum, from superficial to deep. More recent re-evaluations revealed a less distinct, inseparable, sponge-like fascial layer. All authors agree that the superficial dermal layer is probably the optimal location for the injection of volumizing materials because it is free of anatomical structures. Many methods for harvesting, preparing, and injecting fat grafts to the dorsum of the hand have been described in the past three decades. Both filler and fat-graft procedures are performed on an ambulatory basis under local anesthesia. Good results with low postoperative and long-term complication rates and high patient satisfaction have been reported.

## Introduction and background

Though long neglected in the pursuit to look younger, the hands are the most visible part of the body after the face and neck [1], and can serve, by themselves, as a good measure of one’s age [2,3]. In recent years, the upsurge in the popularity of facial rejuvenation procedures in all sectors of the population and the growing armamentarium of treatments have prompted an increase in the demand for hand rejuvenation [4], reaching up to 60%, according to isolated reports [1,5].

The skin on the hands is thin and delicate, and it is prone to developing wrinkles and age spots as we age. The hands are also prone to volume loss, which can contribute to the appearance of wrinkles and creases. In addition, the skin on the hands may become less elastic and less able to bounce back after being stretched or pulled. As the skin loses elasticity and becomes more translucent, the veins, joints, and tendons appear more prominent [3], and the bones become more noticeable [6-8]. These changes are due to intrinsic factors, including loss of collagen and elastin fibers, loss of subcutaneous fat, and bone and muscle atrophy. Extrinsic factors, such as exposure to the sun or household cleaning products, contribute to the damage, causing rhytids, tactile roughness, and changes in pigmentation, including lentigines, actinic keratosis, and seborrheic keratosis [6-8]. In the clinical setting, physicians use the five-point photonumeric Merz Hand Grading Scale (MHGS, Table 1) to rapidly and reliably assess the appearance of the dorsum of the hand based on volume loss, a common sign of aging.

**Table 1:** Merz Hand Grading Scale This Merz Hand Grading Scale is a publicly available and validated scale to grade dorsal hand appearance. This table was created by the authors and not copied from any specific source. Sources used to compile this table have been cited. Source: [9-11]

Grade	Description
0	No loss of fatty tissue
1	Mild loss of fatty tissue; slight visibility of veins
2	Moderate loss of fatty tissue; mild visibility of veins and tendons
3	Severe loss of fatty tissue; moderate visibility of veins and tendons
4	Very severe loss of fatty tissue; marked visibility of veins and tendons

The MHGS consists of five categories, which are scored on a scale from 0 to 4 based on the severity of volume loss. A higher score indicates greater aging. The MHGS has been validated in both photographic and live assessments [9-11]. The currently available options for restoring volume loss in aging hands are autologous fat injection and injectable dermal fillers such as polymethylmethacrylate (PMMA), calcium hydroxyapatite (CaHA), hyaluronic acid (HA), poly-L-lactic acid (PLLA), polycaprolactone (PCL), collagen, and silicone [4,6,12-19].

## Review

Anatomy

Accurate anatomic knowledge is essential for practitioners who work with dermal fillers and fat grafts. This is particularly true when it comes to the dorsum of the hand. In order to identify the optimal locations for injecting dermal fillers and placing fat grafts, practitioners must have a thorough understanding of the anatomy of the dorsum of the hand.

In his guide to the management of hand infections, revised in 1939, Kanavel provided one of the earliest descriptions of the anatomy of the dorsum of the hand [20]. He reported that there are two main layers of tissue present on the dorsum of the hand. The first layer is a superficial fascia, which is located over the extensor tendons. The second layer is a deep fascia, which lines the interosseous muscles and metacarpals. Additionally, he noted that the skin on the dorsal hand is thin and delicate. This thinness of the skin makes it more susceptible to injury and damage. Furthermore, he described that the skin of the dorsal hand contains numerous sensory receptors that allow us to feel touch, pressure, and temperature. These receptors are important for fine motor control and dexterity. Kanavel's description of the anatomy of the dorsal hand is still considered to be accurate and is widely used in the field of hand surgery [20,21]. In a cadaveric study several years later, Anson et al. conducted further research on the fasciae of the dorsum of the hand [21]. They found that the superficial and deep fasciae were not just a single layer but were actually divided into two layers each. The superficial and deep fascial layers were separated by an areolar plane, which is a thin layer of connective tissue that surrounds blood vessels and nerves. This finding provided a more detailed understanding of the anatomy of the fasciae on the dorsum of the hand and is important to consider when injecting dermal fillers and placing fat grafts.

It was only in 2010 that Bidic et al. re-examined the anatomy of the dorsum of the hand from the perspective of hand rejuvenation with fat grafts [5]. Cadaveric samples or fresh cadaveric hands were evaluated histologically, with duplex ultrasound (to assess lamination), and by oxide injection (to assess the vascularity of perforating septa). The results showed that the dorsum consisted of three fatty laminae - superficial, intermediate, and deep - separated by three fascial layers. The intermediate dorsal lamina was traversed by dorsal veins and sensory nerves, and the deep lamina, by extensor tendons; additionally, septal adhesions containing perforating vessels extended from the deep arch to the dermis [8-12]. The superficial dorsal lamina, one of the layers found in the superficial fascia, had no accompanying structures. This means that it does not contain any nerves, blood vessels, or other sensitive structures. Its main role is to cushion and protect the underlying structures of the hand. Given that the superficial dorsal lamina is relatively free of important structures, it was considered the optimal location for fat graft injection [5]. Injecting the fat grafts in this area would minimize the risk of damage to sensitive structures and ensure the best possible outcome for the patient.

Thereafter, Lefebvre-Vilardebo et al. conducted a study on filler injection, using a similar design [22]. They found that the fascial plane between the dermis and tendons, which is the area where the filler is typically injected, measured between 0.3 and 2.2 mm in thickness. They also found that this fascial plane had a three-dimensional sponge-like framework. This framework is important because it contains the veins, which are located at different levels within it. This knowledge is important for practitioners to identify the optimal location for injecting dermal fillers as well as to avoid complications such as injecting into the wrong plane and causing injury to the veins [22]. They used a technique called the Scrape Skin Threading Technique to study the optimal location for filler injection. This technique involves using a cannula, a thin, hollow tube, to scrape the deep side of the dermis. This allows the researchers to access the deeper layers of the skin and study their properties. By using this technique, they concluded that the undersurface of the dermis, which is the deep side of the dermis, was the optimal location for the deposition of filler. This is because the undersurface of the dermis is relatively free of important structures and has a consistent three-dimensional framework that is less likely to cause complications when injecting a dermal filler.

To identify reasons for the adverse outcomes of hand volumizing procedures, Frank et al. re-evaluated the anatomy of the dorsal hand in cadaveric dissections using fluoroscopic, ultrasound, and computed tomography techniques [23]. In line with the study of Bidic et al. [5], it was found that the proximal two-thirds of the dorsum of the hand had clearly defined anatomical layers. However, in the distal third of the dorsum, the arrangement of these layers was less distinct. The superficial dorsal lamina, located less than 1 mm from the skin, was free of neurovascular structures and strongly compartmentalized by longitudinally oriented septa. These authors, too, concluded that volumizing materials were best injected into the superficial lamina [23]. They coined this space “the dorsal superficial lamina.”

A recent study conducted by Park et al. investigated 21 cadavers to study the anatomy of the dorsal intermediate lamina [24]. A previously unidentified fourth fascia was found in the area, according to the study. The dorsal venous plexus and the dorsal cutaneous nerves are located in a deep compartment, which is where the fourth fascia was discovered to be located. In addition to the newly discovered hyperechoic fascia in the dorsal intermediate lamina, the researchers also employed ultrasound image guidance to identify three hypoechoic laminae and three hyperechoic fascial layers. The anatomy of the dorsal intermediate lamina is better-understood thanks to this finding, which may also have significant consequences for surgical interventions there. Further investigation in this area may shed light on the function of the newly identified fascia and its relevance to clinical practice.

To analyze the fat compartments of the dorsal hand, Zhou et al. injected methylene blue dye into the superficial lamina [25]. The researchers discovered that the proximal portion of the superficial lamina contained three separate fat compartments as a result of their study. Based on their positioning in relation to the radial, middle, and ulnar areas of the hand, these compartments were given the names radial, middle, and ulnar.

The study also showed that the superficial lamina's distal portion was divided into four compartments that were situated in the space between the metacarpophalangeal joints. The researchers discovered that these compartments were structurally diverse from one another and identified their distinctive features.

The findings of this study provide a more detailed understanding of the anatomy of the superficial lamina, which could have important implications for surgical procedures and other medical interventions in the hand. The identification of distinct compartments within the superficial lamina could potentially aid in the diagnosis and treatment of hand-related conditions, as well as contribute to the development of more effective therapies. Further research in this area may also shed light on the function of these compartments and their significance in hand anatomy and physiology.

Current treatment methods

Dermal Fillers

Only two fillers are currently Food and Drug Administration (FDA)-approved for hand rejuvenation: Radiesse® (CaHA; Merz North America, Inc., Raleigh, NC) and Restylane-Lyft® (hyaluronic acid; Galderma Laboratories, Fort Worth, TX). Dermal fillers like CaHA are widely used to enhance the appearance of the hands, which are prone to symptoms of aging such as volume loss, creases, and thinning of the skin. CaHA is created from a mineral that is naturally found in bone tissue, making it a safe and biocompatible product that can be employed in aesthetic procedures. To achieve the best results, some experts recommend diluting the CaHA 1:1 and injecting it into a single site on each hand using a cannula. The overall injection volume recommended for hand rejuvenation is typically 2.5 ml per hand [26]. CaHA is regarded as a filler that lasts for up to a year or longer, with results. Most of the time, the operation is safe and minimally invasive, and recovery time is little to nonexistent. Hyaluronic acid should be injected into the subcutaneous layer at several sites in a five-step procedure, with 0.5 ml injected in each webspace to a total of 2 ml per hand [27]. It's crucial to take a cautious approach and inject tiny amounts of the filler material at various spots while utilizing dermal fillers to revitalize the hands. This method of injection, called "microdroplet," aids in ensuring that the filler is spread uniformly and does not cause lumps or bumps in the skin. The final result can be altered to suit each patient's particular requirements and preferences by injecting only modest amounts of filler material. The risk of overfilling or harming delicate hand tissues, including tendons or nerves, is also diminished by dispersing the injections throughout a number of sites. Although there are reports of an increasing number of complications following the use of dermal fillers [9,23,28], they are considered generally safe for hand rejuvenation when correctly applied [29]. Dermal fillers can provide temporary improvements in the appearance of the hands, but the effects are not permanent. Repeat treatments are typically needed to maintain the desired results.

Fat Grafting

Dermal fillers have grown in popularity as a treatment option for those who want to add volume to their hands, face, or other body parts. While fillers can deliver quick, pleasant results, there are also some side effects like infection, allergic reactions, or uneven filler distribution that could occur when using them. Because fat grafts use the patient's own tissue rather than a manufactured substance to produce the desired look, some studies indicate that they may be a safer and more long-lasting option than dermal fillers. Additionally, fat grafts may have a decreased chance of negative reactions or problems, as well as longer-lasting results [1,2]. The first fat-grafting technique, published by Fournier in the 1980s, and the many subsequent variants thereof, were based on the injection of a large bolus of fat into the dorsum through a single incision that was then spread throughout the rest of the hand by gentle massage. However, the results were unpredictable, variable, and unreliable over the long term [1]. In 1992, Coleman modified the technique by delivering fat in a structured fashion via many minuscule tunnels to maximize the surface area of fat-tissue contact [8]. This resulted in fewer complications and higher patient satisfaction.

Harvesting of autologous fat for grafting is performed under local anesthesia with or without sedation. Usually, a tumescent solution of lidocaine and epinephrine is injected at the donor site. The fat is harvested using a blunt cannula, usually from the abdomen, flank, thigh, or medial knees, and then centrifuged or decanted [4,30]. Butterwick demonstrated that centrifuged fat had better longevity in some patients than non-centrifuged fat during a follow-up of three years [22]. Others, however, reported good results and high patient satisfaction with decantation alone [7] or the use of an operating towel to concentrate the fat [31]. As an alternative to conventional fat grafting techniques, nanografting has been developed. Using a specialized cannula, micro-fat is harvested, which is subsequently emulsified up to 30 times to produce tiny, homogeneous fat particles. Following the pressing of these particles through nylon cloth, a smooth, uniform graft material that may be injected into the desired location is produced. In comparison to previous fat grafting methods, using nanografting may have a number of benefits, including a lower risk of problems, a more realistic-looking outcome, and the ability to focus on more precise parts of the body [32]. It has been found to be useful for the treatment of traumatic scars and burn wounds and under split-thickness grafts [32-34].

Before grafting, the hands are prepared and draped in a sterile fashion and anesthetized with lidocaine or by nerve block at the wrist or fingers; gentle massage is used to disseminate the anesthetizing solution. Between 10 and 30 mL of autologous fat per hand is transferred to 1 ml syringes. The surgeon makes several small incisions in the dorsum in a radiating pattern and injects the fat in a retrograde fashion, depositing small aliquots as the cannula is withdrawn. The number and locations of the incision vary in different studies [2,7,35,36]. Some surgeons advocate injecting the fat into the base of each finger or the radial and ulnar aspects of the fingers [35,37]. Most agree that the hand should appear slightly overfilled and puffy at the end of the procedure. Postoperatively, patients are instructed to keep their hands elevated for 24 to 48 hours and to avoid manual activity for one to two weeks. Some surgeons prescribe antibiotics for up to 10 days [1,4]. As fat graft survival might be as low as 50% in the long term, "overfill" just after treatment may be required [38].

Complications

Although injectable dermal fillers are typically thought to be safe and efficient for skin rejuvenation, there is a chance that the procedure could have some short-term negative effects. Bruising and edema, or swelling, in the treated area are two of the most typical side effects. These side effects are normally brief, mild, and go away on their own in a few days or weeks.

Depending on the filler used, the injection technique, the person's anatomy, and the healing process, bruising and edema might vary in size and duration. Through careful pre- and post-treatment care, such as avoiding blood-thinning drugs or administering cold compresses to the treated area, these side effects may, in some cases, be reduced. Potential adverse long-term sequelae include swelling, sensory dysfunction, and foreign body granuloma formation, sometimes permanent. Autologous fat grafting is associated with a few complications, which may include infection of the hand and harvest site, transient digital numbness, temporary dysesthesia, cyst formation, fat necrosis, and reabsorption of the grafted fat [12]. In one study of a hand rejuvenation technique that incorporated lipotransfer to the fingers, 72% of patients could not wear their rings after the procedure because of the increased volume of their fingers; however, 68% of them were not bothered by this side effect [35]. It is not uncommon for injectable treatments, including dermal fillers and fat grafting, to cause temporary changes in the size or shape of the treated area. Indeed, a small case series reported no major intraoperative or postoperative complications other than transient edema and ecchymosis [2,25,35]. In a review of their 15-year experience with fat grafting in 65 patients, Fantozzi reported no permanent or long-term complications over one year of follow-up [7]. Accordingly, two recent systematic reviews of hand rejuvenation using fat grafting or dermal fillers did not find reports of major complications in any study. Minor complications were mainly edema and pain and were only temporary. The authors concluded that overall, hand rejuvenation procedures are safe and efficacious, and rates of patient satisfaction are high [39,40].

Discussion

Dorsal hand rejuvenation is a cosmetic procedure that has gained popularity in recent years due to the increasing focus on achieving a more youthful appearance. In addition to being performed as a solitary procedure, dorsal hand rejuvenation is also commonly used as an adjunct to face and neck rejuvenation treatments.

As the demand for hand rejuvenation procedures has grown, there has been a corresponding increase in research on understanding the anatomy of the dorsal hand and developing useful assessment tools to evaluate the appearance of the hands. The skin on the dorsum of the hand is prone to developing wrinkles and age spots due to intrinsic and extrinsic factors. Furthermore, volume loss due to aging or weight loss can contribute to the appearance of wrinkles and creases.

The two main techniques used for dorsal hand rejuvenation are autologous fat transfer and dermal fillers. Autologous fat transfer involves harvesting fat from one part of the patient's body and injecting it into the dorsum of the hand. Dermal fillers are synthetic materials that are injected into the dorsum of the hand to restore volume loss. Both of these techniques can be performed under local anesthesia, and patients have reported high levels of satisfaction with the outcomes.

**Figure 1:**
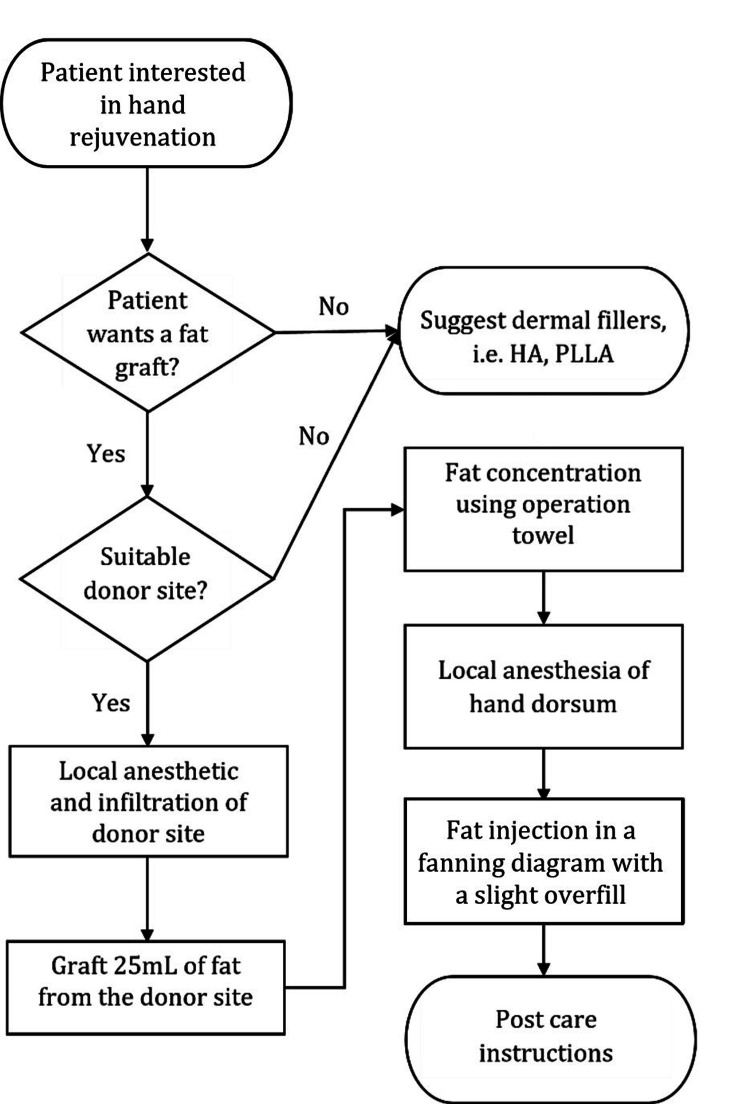
Suggested Algorithm for Hand Rejuvenation HA – hyaluronic acid; CaHA – calcium hydroxyapatite

To help guide practitioners in the decision-making process for hand rejuvenation, an algorithm has been developed. This algorithm takes into account patient preferences, the degree of skin laxity, and the presence of volume loss, among other factors (Figure 1).

We believe autologous fat grafting and dermal filler injection for hand rejuvenation should be included in the armamentarium of dermatologists and plastic surgeons.

## Conclusions

The popularity of rejuvenation procedures and the accessibility of injectable therapies have led to an increase in the demand for hand rejuvenation in recent years. Due to both intrinsic and external factors, the skin of the hands is prone to acquiring wrinkles and age spots, and volume loss can exacerbate the appearance of wrinkles and creases. Injectable dermal fillers and autologous fat injections are a few possibilities for replacing the volume lost in aging hands. Practitioners who work with dermal fillers and fat grafts on the dorsum of the hand need a thorough understanding of anatomical structure. The anatomy of the dorsum of the hand has been widely studied in cadaveric and clinical studies, providing a thorough understanding of the superficial and deep fasciae, fatty laminae, and vascularity of the area. The optimal location for injecting dermal fillers and placing fat grafts is the superficial dorsal lamina, which has no accompanying structures and is located less than 1 mm from the skin. It is crucial for practitioners to identify this location to minimize the risk of damage to sensitive structures and ensure the best possible outcome for the patient. The five-point photonumeric Merz Hand Grading Scale is a useful tool for assessing the effectiveness of these treatments, but more research is needed to determine their long-term effects on the anatomy of the dorsum of the hand. Understanding the anatomy of the dorsum of the hand is essential to prevent the adverse outcomes of hand volumizing procedures.
